# Cardiopulmonary deconditioning and plasma volume loss are not sufficient to provoke orthostatic hypertension

**DOI:** 10.1038/s41440-024-01710-x

**Published:** 2024-05-23

**Authors:** J.-N. Hoenemann, S. Moestl, L. de Boni, F. Hoffmann, M. Arz, L. Berger, D. Pesta, K. Heusser, E. Mulder, S. M. C. Lee, B. R. Macias, J. Tank, J. Jordan

**Affiliations:** 1 German Aerospace Center—DLR, Institute of Aerospace Medicine, Cologne, Germany; 2 University of Cologne, Faculty of Medicine and University Hospital Cologne, Clinic III for Internal Medicine, Kerpener Str. 62, 50937 Cologne, Germany; 3 KBR, Houston, TX USA; 4 NASA Johnson Space Center, Houston, TX USA; 5 Medical Faculty, University of Cologne, Cologne, Germany

**Keywords:** Orthostatic hypertension, Weightlessness, Bedrest, Deconditioning, Sympathetic nervous systems

## Abstract

Orthostatic hypertension, defined by an increase of systolic blood pressure (SBP) of ≥20 mmHg upon standing, harbors an increased cardiovascular risk. We pooled data from two rigorously conducted head-down tilt bedrest studies to test the hypothesis that cardiopulmonary deconditioning and hypovolemia predispose to orthostatic hypertension. With bedrest, peak VO_2_ decreased by 6 ± 4 mlO_2_/min/kg (*p* < 0.0001) and plasma volume by 367 ± 348 ml (*p* < 0.0001). Supine SBP increased from 127 ± 9 mmHg before to 133 ± 10 mmHg after bedrest (*p* < 0.0001). In participants with stable hemodynamics following head-up tilt, the incidence of orthostatic hypertension was 2 out of 67 participants before bedrest and 2 out of 57 after bedrest. We conclude that in most healthy persons, cardiovascular deconditioning and volume loss associated with long-term bedrest are not sufficient to cause orthostatic hypertension.

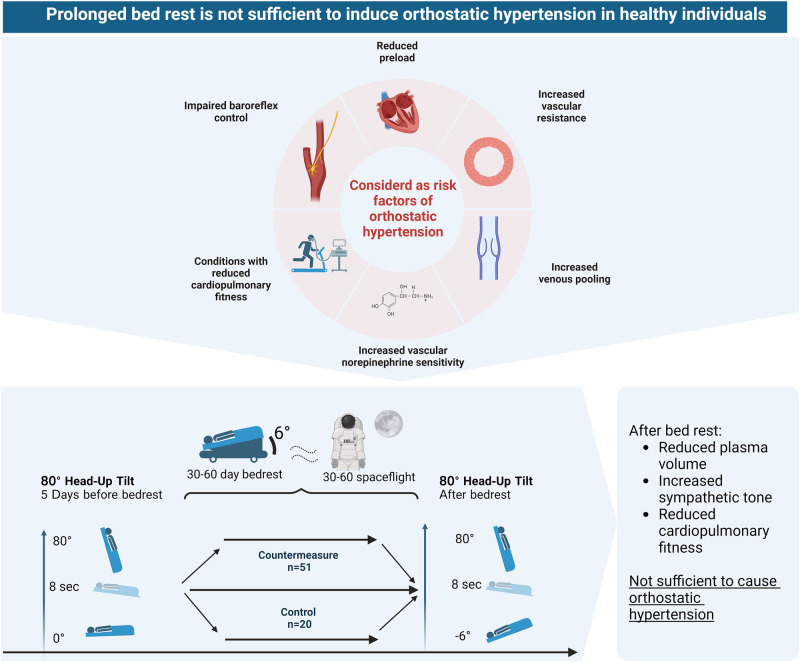

## Introduction

Orthostatic hypertension, defined as sustained ≥20 mmHg systolic blood pressure (SBP) increase when standing, heralds increased cardiovascular risk [1–3]. Even smaller orthostatic SBP increases are associated with excess cardiovascular risk [4]. Orthostatic hypertension is mediated through sympathetic overactivation [5], however, the mechanisms driving the response are not fully understood. Central hypovolemia through volume deficiency or excess venous pooling has been implicated [6]. Moreover, observations in rare conditions associated with orthostatic SBP increases suggest that impaired baroreflex counter-regulation may be involved [7]. Conditions that predispose to orthostatic hypertension like advanced age, obesity, or postural tachycardia syndrome (POTS) are often associated with reduced cardiopulmonary fitness [8, 9]. Increased cardiopulmonary fitness is associated with increased plasma volume, improved baroreflex function, and attenuated sympathetic activity [10]. Conversely, deconditioning during head-down tilt bedrest, an established model for weightlessness, reduces plasma volume, worsens baroreflex function, and increases sympathetic activity [11]. We hypothesized that cardiopulmonary deconditioning and hypovolemia through head-down tilt bedrest increases the likelihood of experiencing an orthostatic SBP increase and that the extent of cardiopulmonary deconditioning and plasma volume reduction predicts the response.

## Methods

### Study participants and protocol

We pooled data from two head-down tilt bedrest studies conducted at the: envihab facility of the German Aerospace Center (DLR). The AGBRESA study included 24 participants (8 women/16 men, 33.3 ± 9 years) who were assigned to 60-day head-down tilt bedrest. Participants were distributed to intervention groups, which were submitted to 30 min/day continuous or intermittent artificial gravity training on a short-arm centrifuge, or a control group. The SANS-CM study included 47 participants (20 women/27 men, 35 ± 9 years) undergoing 30 days head-down tilt bedrest who were randomized to a negative control group, a positive control group (sitting 6 h/day), a group submitted to 6 h/day of −25 mmHg daily lower body negative pressure, and a group in which 60 min of head-down cycling was followed by 6 h venous thigh cuff occlusion (50 ± 5 mmHg) on 6 days/week. Participants were healthy on no medications. During the study, they were on controlled sodium / isocaloric diets with standardized fluid consumption. We obtained written informed consent before study entry. Both studies were approved by the local ethics committee (Northrhine Medical Association).

### Cardiovascular assessment

We conducted 80° head-up tilt table testing 5 days before and immediately after bedrest. Following 10 min supine rest, we acquired three brachial blood pressure measurements over 5 min. Following the head-up tilt, we measured blood pressure every 2 min after a 1 min hemodynamic stabilization period. We computed SBP variability in the low-frequency range from continuous finger blood pressure supine and following hemodynamic stabilization at 80° head-up tilt [11]. We determined heart rate from continuous three-channel ECG over 60–300 s supine and following hemodynamic stabilization at 80° head-up tilt. Participants with missing measurements or rapid presyncope were excluded from analysis. We determined plasma volume using carbon monoxide rebreathing before and after bedrest [12]. Maximum oxygen uptake was measured during cardiopulmonary exercise testing at baseline and after bedrest.

### Statistics

All values are presented as mean ± standard deviation. *P* < 0.05 indicates statistical significance. All analyses were performed in GraphPad Prism (Software version 10.0.2). We compared parameters by a paired t-test and Pearson correlation (*r* = 0.10–0.29 weak correlation, *r* = 0.30-0.49 moderate correlation; *r* ≥ 0.5 = strong correlation). All data were collected through the International Standard Measures protocol and shared between DLR and NASA. Data supporting our results are available from the corresponding author upon reasonable request.

## Results

In the pooled sample comprising participants in control groups and participants submitted to countermeasures, maximal oxygen uptake decreased from 36 ± 8 mlO_2_/min/kg before to 30 ± 7 mlO_2_/min/kg following bedrest (*p* < 0.0001). Plasma volume decreased by 367 ± 348 ml with bedrest (*p* < 0.0001). Control group and pooled countermeasure groups showed reductions in cardiopulmonary fitness and plasma volume (Table 1).

**Table 1:** Response to head-down tilt bedrest

Parameter	Group	Before bed rest	After bed rest	*p*-value
Peak VO_2_ (ml O2/min/kg)	All	35.55 ± 7.62	29.8 ± 6.98	<0.0001
	Countermeasure	35.48 ± 7.91	30.8 ± 7.28	<0.0001
	Control	35.64 ± 7.06	27.2 ± 5.47	<0.0001
Plasma volume (ml)	All	3047 ± 489.8	2676 ± 400.6	<0.0001
	Countermeasure	3082 ± 500.7	2694 ± 409.4	<0.0001
	Control	2958 ± 461.8	2629 ± 383.8	<0.0001
Systolic blood pressure supine (mmHg)	All	127 ± 9	133 ± 10	<0.0001
	Countermeasure	127 ± 9	132 ± 10	<0.0001
	Control	129 ± 7	135 ± 10	0.0022
Systolic blood pressure standing (mmHg)	All	129 ± 12	135 ± 13	<0.0001
	Countermeasure	129 ± 11	135 ± 13	<0.0001
	Control	129 ± 14	134 ± 14	0.1962
Diastolic blood pressure supine (mmHg)	All	79 ± 9	81 ± 8	0.0478
	Countermeasure	80 ± 10	79 ± 8	0.9946
	Control	77 ± 6	86 ± 8	<0.0001
Diastolic blood pressure standing (mmHg)	All	85 ± 9	87 ± 12	0.184
	Countermeasure	85 ± 9	85 ± 12	0.974
	Control	83 ± 9	91 ± 12	0.007
Low-frequency systolic blood pressure supine (mmHg²)	All	5.29 ± 4.6	9.07 ± 7.33	<0.0001
	Countermeasure	5.49 ± 5.17	8.51 ± 7.31	0.0091
	Control	4.73 ± 2.5	10.58 ± 7.37	0.0011
Low-frequency systolic blood pressure standing (mmHg²)	All	27.18 ± 33.07	36.45 ± 35.28	0.0608
	Countermeasure	37.89 ± 36.35	35.92 ± 36.97	0.0375
	Control	25.28 ± 22.73	38.07 ± 30.56	0.1917
Heart rate supine (bpm)	All	68 ± 11	79 ± 14	<0.0001
	Countermeasure	67 ± 11	76 ± 13	<0.0001
	Control	71 ± 10	88 ± 12	<0.0001
Heart rate standing (bpm)	All	92 ± 15	120 ± 19	<0.0001
	Countermeasure	90 ± 15	117 ± 19	<0.0001
	Control	98 ± 16	129 ± 4	<0.0001

Mean values and standard deviation of all participants (all), pooled data from participants who underwent a countermeasure during bed rest, and pooled control groups before and after bed rest including *p* values (paired *t*-test)

Supine blood pressure was 127 ± 9/79 ± 9 mmHg before and 133 ± 10/81 ± 8 mmHg mmHg after bedrest (*p* < 0.0001/*p* = 0.0478). During 5 min standing, blood pressure changed 2 ± 8 mmHg/5 ± 5 mmHg before bedrest and 3 ± 8/6 ± 10 mmHg after bedrest (*p* = 0.5825/*p* = 0.7548). Supine heart rate increased from 69 ± 11 bpm before to 79 ± 15 bpm after bedrest (*p* < 0.0001). The orthostatic heart rate increase was 21 ± 12 bpm before and 39 ± 16 bpm after bedrest (*p* < 0.0001).

**Fig. 1:**
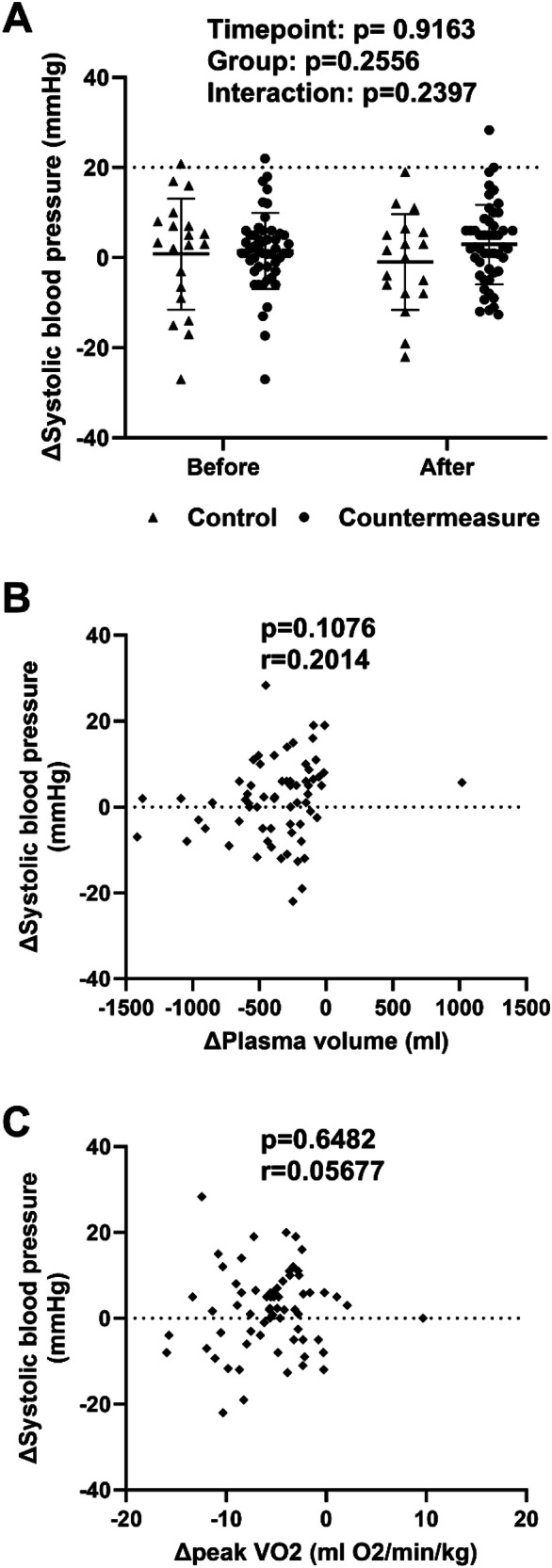
Individual differences in systolic blood pressure responses when changing from the supine to 80° head-up tilt position before and after bedrest (**A**). We averaged up to three upper arm blood pressure measurements while supine and in first 5 min at 80° head-up tilt following hemodynamic stabilization of 1 min. The dashed line marks the diagnostic threshold for orthostatic hypertension. Correlation between the change in the systolic blood pressure response to standing and changes in plasma volume (**B**) or maximal oxygen uptake (**C**) with bedrest deconditioning

Orthostatic hypertension occurred in 2 out of 67 participants before bedrest and in 2 out of 57 participants after bedrest. Presence or absence of countermeasures during bedrest did not affect the likelihood of experiencing orthostatic hypertension (Fig. 1A). Two participants showed > 20 mmHg SBP reductions with head-up tilt before and one participant after bedrest. A >10 mmHg diastolic blood pressure reduction with head-up tilt occurred in none of the participants before and in three participants after bedrest. Thus, orthostatic hypotension occurred in two before and in four participants after bedrest.

With bedrest, low-frequency SBP oscillations increased while supine and tended to increase with standing (Table 1)

The difference in the SBP change with standing between before and after bedrest did not correlate with plasma volume or peak VO_2_ changes (Fig. 1B, C).

## Discussion

Cardiovascular deconditioning and volume loss associated with head-down tilt bedrest while changing cardiovascular autonomic control towards sympathetic activity are not sufficient to elicit orthostatic hypertension in healthy persons. While earlier studies suggested that volume loss can cause or exacerbate orthostatic hypertension, we did not observe a relationship between magnitude of plasma loss during bedrest deconditioning and orthostatic SBP responses.

Participants in our study experienced cardiovascular deconditioning during head-down tilt bedrest while other potentially confounding variables remained unchanged. Participants were on a sodium-controlled diet and body weight remained stable throughout the study. Both, sodium intake and caloric balance are known to affect cardiovascular autonomic control. Moreover, we confirmed the degree of cardiovascular deconditioning using cardiopulmonary exercise testing and directly measured plasma volume.

Previously, orthostatic hypertension was attributed to excessive sympathetic activation provoked by hypovolemia and increased venous pooling in specific patient populations [6, 7]. Venous compression garments or volume loading paradoxically attenuated orthostatic hypertension. Our observation that changes in plasma volume did not increase the likelihood of experiencing orthostatic hypertension suggests that additional mechanisms are required to develop orthostatic hypertension.

Mechanistically, orthostatic hypertension could result from sympathetic overactivation, increased vascular sensitivity to norepinephrine, or both combined. Orthostatic hypertension in individuals with the POTS, which is characterized by hyperadrenergic symptoms while standing, supports the idea [13]. For sustained blood pressure increases with standing, baroreflexes have to be reset to higher blood pressure or fail entirely. In fact, rare patients with genetic brachydactyly and hypertension or with afferent baroreflex failure, conditions associated with impaired baroreflex counter-regulation, are susceptible to orthostatic hypertension [7]. In our study, supine and upright heart rates and low-frequency SBP oscillations increased with bedrest deconditioning, which indirectly indicates cardiovascular sympathetic activation. Previously, head-down bedrest was shown to increase sympathetic vasomotor tone directly measured through microneurography [14]. However, the increase in sympathetic activity in our study was not associated with orthostatic hypertension. Possibly, sympathetic activation was the proper response to maintain blood pressure in the face of cardiovascular deconditioning and hypovolemia. Yet, intact baroreflex counter-regulation prevented an excessive blood pressure increase.

The major limitation of our study is that we only submitted healthy persons to bedrest deconditioning and plasma volume loss. Our findings cannot be simply extrapolated to persons with cardiovascular disease. Moreover, we pooled two bedrest studies which varied in duration. Finally, some study participants were submitted to countermeasures, such as lower body negative pressure or centrifugation, which may have attenuated cardiovascular deconditioning. However, plasma volume changes, cardiopulmonary deconditioning, and orthostatic blood pressure responses did not differ qualitatively between participants in the control group and in the pooled countermeasure group.

We conclude that bedrest deconditioning in healthy younger persons is not sufficient to elicit orthostatic hypertension despite substantial plasma volume reductions and sympathetic activation. Additional factors like impaired baroreflex control and changes in vascular structure, which augment sympathetic influences on blood pressure, may be required. In fact, conditions predisposing to orthostatic hypertension like advanced age, obesity, type 2 diabetes mellitus, and essential hypertension are also associated with impaired baroreflex control and changes in vascular structure [8]. Given profound effects of cardiopulmonary fitness on plasma volume and cardiovascular autonomic control, effects of training on orthostatic hypertension deserve attention. Finally, our study supports the robustness of orthostatic hypertension as cardiovascular biomarker because transient changes in cardiovascular control and volume status did not produce “false positive” responses.
